# Teleworking—An Economic and Social Impact during COVID-19 Pandemic: A Data Mining Analysis

**DOI:** 10.3390/ijerph19010298

**Published:** 2021-12-28

**Authors:** Grigore Belostecinic, Radu Ioan Mogoș, Maria Loredana Popescu, Sorin Burlacu, Carmen Valentina Rădulescu, Dumitru Alexandru Bodislav, Florina Bran, Mihaela Diana Oancea-Negescu

**Affiliations:** 1 Academy of Economic Studies of Moldova (University), Kishinev MD-2005, Moldova; belostecinic@yahoo.com; 2 Department of Economic Informatics and Cybernetics, Faculty of Economic Cybernetics, Statistics and Informatics, Bucharest University of Economic Studies, 010374 Bucharest, Romania; mogos.radu@gmail.com; 3 Faculty of Marketing, Bucharest University of Economic Studies, 010374 Bucharest, Romania; maria.popescu@mk.ase.ro; 4 Faculty of Administration and Public Management, Bucharest University of Economic Studies, 010374 Bucharest, Romania; 5 Faculty of Agrifood and Environmental Economics, Bucharest University of Economic Studies, 010374 Bucharest, Romania; carmen-valentina.radulescu@eam.ase.ro (C.V.R.); florina.bran@eam.ase.ro (F.B.); 6 Department of Economics and Economic Policies, Faculty of Theoretical and Applied Economics, Bucharest University of Economic Studies, 010374 Bucharest, Romania; alex.bodislav@ase.ro; 7 Department of Financial and Economic Analysis and Valuation, Faculty of Accounting and Management Information Systems, Bucharest University of Economic Studies, 010374 Bucharest, Romania; mihaela.oancea@cig.ase.ro

**Keywords:** teleworking, economic and social factors, data mining analysis

## Abstract

The health crisis generated by the COVID-19 pandemic has induced, among other things, an increase in the importance of remote work or teleworking (TL) in the current period. The objective of this research is to identify the economic and social impact of telework in changing the behavior of employees in Romania. The research was conducted approximately one year after the onset of the pandemic until the beginning of the vaccination period in Romania. The research proposed includes three main directions of analysis of the extracted data, which are related to telework efficiency, this being considered one of the most important indicators for a company. In order to obtain conclusive results, we used a mixed methodology, combining results obtained through a survey based on a self-administered electronic questionnaire, with a data mining analysis. Detailed analysis of the groups identified based on work efficiency allowed us to highlight the most common employee profiles. This analysis was doubled by a second classification experiment, which provided us a more detailed analysis of the groups identified based on job satisfaction and highlighted the most common employee profiles. The expansion of telework in various economic areas is a result of adaptation to the new economic and social conditions caused by the COVID-19 pandemic.

## 1. Introduction

Telework or remote work has been a hot topic in current studies, probably due to the COVID-19 pandemic. Thus, more and more current research attaches more and more importance to understanding the specifics of remote work or teleworking (TL) in the current period. In this context, our research was also conceived. This was done about a year after the beginning of the pandemic and before the start of the vaccination campaign in Romania. Both studies and the reality show that employees work differently than they did a decade ago. However, the figures suggest that they do not necessarily work much longer. It is found that there will always be workaholics, that is, individuals who, for a variety of reasons, choose to work overtime. Some employees work long hours due to work overload. Others are happy to do this because they are truly involved in what they are doing [1]. During the COVID-19 pandemic, many businesses were forced to close, and some employees were given the opportunity to telework for the first time.

Disparities in the number of people who stayed in their jobs may have contributed to some COVID-19 disparities [2].

Many things had to be done differently in a forced manner with the start of epidemic and TL, and time was too short to allow all parties concerned (such as employees, employers, and the state) to adjust. This adaptation was made in a forced manner taking into account the short time available. The employee must adjust to a variety of factors, including location, modality of accomplishing own tasks, communication with colleagues and superiors, and modality of presenting outcomes of own activities. On the other side, employees’ limited interaction with family and friends contributes to their mental health difficulties, an interaction that is often a refuge for work problems.

Even certain security health precautions were suspended during the pandemic, according to a survey conducted by PricewaterhouseCoopers (PwC) on 300 firms, 45% of enterprises will not urge workers to return to work after the epidemic, and 21% are considering a hybrid strategy (TL and work office) [3]. As a result, while TL was a forced health precaution intended to stop the virus from spreading during the pandemic, it can become a way of life for certain people after the epidemic and a realistic choice for others who want to return to work.

## 2. Literature Review

Research in this area has focused on equal opportunities for workers trying to explain and combat, through strategies, the digital division [4]. On the other hand, it tries to provide a rational answer to the question of whether telework can be an opportunity for sectors where there is insufficient labor force in the territory [5]. A study of telework in the information age makes an exhaustive analysis of the literature and finds that the generic term “teleworker” refers to someone who works in a place other than where the results of work using ICT are needed. It should be noted that much of the literature describes or prescribes changes in working practices that are facilitated by technology [6].

Data mining is considered a new technological reality that requires appropriate tools to exploit the mass elements of information, that is, data. It is something that may seem paradoxical, but it is in fact considered that most of the time, it tells us that we cannot get meaningful information from such an abundance of data. The importance of this method is argued, given that simple data analysis tools can result in valuable information at the end of the process: often an elementary scatter plot can provide useful guidance, although formal analysis can be much more sophisticated. Moreover, the image can change dramatically: many of the simple tools used lose their effectiveness [7].

On the other hand, the concept of raw dataset is a new mathematical approach to counter inaccuracy, vagueness, and uncertainty in data analysis. A possible starting point of the gross set philosophy is the assumption that each object of interest can be associated with certain information, data, or knowledge. As a consequence of this definition of the raw dataset, it was stated that each raw set has boundary line elements, that is, elements that cannot certainly be classified as members of its set or complement. In other words, it is found that borderline cases cannot be properly classified using available knowledge. Thus, raw sets could be seen as a mathematical model of vague concepts [8]. 

Regarding Data Mining, it is a computer/computer-based system dedicated to scanning huge data warehouses, generating information, and discovering knowledge. It is mentioned that a significance of the traditional mining term would influence the data mining lands. By similarity, instead of looking for natural minerals, the target in this case is knowledge. Data mining would aim to discover data patterns, organize information about hidden relationships, structure rules of association, estimate the values of unknown elements to classify objects, compose homogeneous groups of objects, and reveal many types of discoveries that are not easily produced by a classic computer/computer-based system. Therefore, the results of data mining would be a valuable support for decision making [9]. 

Recent research considers that the exploitation of association rules is found to be one of the most popular methods of data analysis that could discover associations within the data. Algorithms for extracting association rules can be applied to different datasets, due to their practical utility. As it was found that little attention was given to the application of association mining techniques to analyze the data in the questionnaire, researchers first identified the different types of data that may appear in a questionnaire. Then, they introduced the issue of mining the data in the questionnaire and defined patterns of rules that can be extracted from the data in the questionnaire. It has been developed on the basis of a unified approach to fuzzy techniques, so that all different types of data can be treated in a uniform manner. After that, an algorithm was developed to discover the unclear association rules in the questionnaire dataset. Finally, they evaluated the performance of the proposed algorithm, and the results obtained by them indicated that the method would be able to find interesting association rules that have never been found by previous mining algorithms [10].

Dissemination of recent research has shown that in recent years, data extraction and text extraction techniques have been frequently used to analyze questionnaires and review data. Data mining techniques, such as association analysis and cluster analysis, would be used for marketing analysis, as they could uncover relationships and rules that are hidden in huge numerical data. On the other hand, text retrieval techniques, such as keyword retrieval and opinion retrieval, could be used for questionnaire analysis or text review, as they could help to investigate consumer opinion in the text data. However, data extraction tools and text extraction tools could not be used in a single environment [11].

On the other hand, data preparation is a fundamental stage of data analysis. This is because while a lot of low-quality information may be available in various data sources and on the Web, many organizations or companies may be interested in how to turn data into clean forms that could be used for profit obtained. It was found that this objective generates an urgent need for data analysis aimed at cleaning up raw data [12].

Hirji argued that data mining can become a class of analytical techniques that goes beyond statistics and seek to examine substantial amounts of data [13]. His study provided a new perspective on how data should be extracted. He suggested a few practical recommendations for planning, managing, and executing projects that managers and practitioners could follow in data mining implementation projects. He found that data mining seems to follow a more elaborate set of steps than previously reported. The project no longer focused on building a production data mining application, but on determining business objectives, data preparation, data auditing, interactive data extraction and analysis of results, back-end data extraction, synthesis, and presentation of results. 

The COVID-19 pandemic has impacted a wide range of industries, including micro, small, and medium-sized enterprises (SMEs) [14]. In this context, research is being conducted to determine the level of sustainability of SMEs in cities, as well as the efforts that can be made to help SMEs survive and gain a competitive advantage in their respective industries. As a result, they established a theoretical foundation on which the governments of various nations might build policies to promote the development of SMEs. According to the findings of their research, improvements in internal processes did not have an impact on the degree of sustainability of SMEs; in addition, improvements in internal processes did not have an impact on the profile of SMEs and external environmental support.

Additionally, there are scholarly studies that demonstrate that corporations that utilize efficient e-leadership (such as teleworking) perceive it as an opportunity [15]. Telework is considered to be advantageous both for the productivity of companies and for the environment and for people who work remotely. On the other hand, the same studies show that a traditional management of companies could lead to certain risks when working in remote work environments implies that managers have to adjust the structure of companies (e.g., to reduce hierarchical steps, to develop new skills to establish a strong and trusting relationship with employees, maintain competitiveness and maintain a real concern for the well-being of their employees [16]). Consequently, telework, according to recent studies, is characterized by both advantages and disadvantages for employers and employees. Of course, these characteristics also vary due to the many forms of teleworking: from occasional work outside the office, to freelance in collaboration centers, to work on the road [17]. 

Dewi and colleagues (2020) estimated the pandemic’s COVID-19 score, based on the doubling time, and discovered that it was closely related to the health security category. More than half of the nations in this study’s dataset (117 countries) responded disproportionately to the pandemic policy on COVID-19, according to the findings. In the area of Global Health Security, six nations were recognized as the best-trained, with a medium to high pandemic score. As a result, in response to the pandemic policy, they were identified as insufficient and minimal responses. Despite the fact that Brazil and South Africa were among the countries with the greatest levels of health security, both countries had a significant rise in COVID-19 infections [18].

According to Omrani, even though the COVID-19 pandemic has affected all of humankind, straining health systems, economies and governments around the world, and that one of the responses to the pandemic has been a major global effort to collect, analyze, and address publicly available data, many of the existing public datasets regarding COVID-19 are aggregated at country level and tend not to bridge the gap. However, these characteristics are regarded as critical in the research of SARS-CoV-2 transmission and severity. Their research introduced a new collection of data for the European Union at the level of small regions (NUTS3), including socio-demographic, economic, public policy, health, pollution, and environmental variables, to aid in assessing the causes and effect of the COVID pandemic. This data collection might be used to track mortality and COVID-19 infections at the subnational level, as well as conduct analyses that can assist influence future policy decisions [19].

Research prior to the COVID-19 pandemic has tried to find and explain the reasons for working from home. Moreover, they tried to highlight ways to create and maintain borders at home/work, isolation problems, distractions and temptations faced by home workers. They then emphasized labor handling and gender differences. Some research addresses six areas of research: reasons for working at home, creating and maintaining home/work boundaries, isolation challenges, diversions and temptations faced by home employees, work addiction, and gender disparities. Their results show that white-collar workers usually work from home to prevent work or family conflicts. However, isolation, diversions and temptations at home and work addiction are real issues, but they may have been overestimated. [20]. Notorious is the finding of researchers who believe that the problems of creating and maintaining borders at home/work, isolation, distraction and temptations at home and work practice are real but also showed evidence that they could have been exaggerated in previous writings on telework or work from home [21]. The approach to telework before the pandemic has been polarized, studies have shown more than a decade ago, with the literature highlighting optimistic and pessimistic reports about the impact of increasing the working arrangements of the alternative location [5]. The research results of that period provide us with qualified support for the more optimistic perspective of work from home. The idea is supported by the finding that working hours at home are positively associated with employees’ well-being, job satisfaction and life, but also negatively associated with exhaustion and stress. However, it should be noted that for professionals in the knowledge-based industry, working from home can act as an antidote to the stress and tension caused by office work. Studies also show that improved welfare of home workers can come at a price. One could be to undermine the desire of organizations to invest in the training and development of teleworkers and would have a negative impact on career progression [22].

Research in the field of telework has tried to answer questions such as who participates in telework, why they do it and what happens when they do it. The answers were not long in coming and inspired research in disciplines ranging from transportation and urban planning to ethics, law, sociology and organizational studies [23].

The COVID-19 pandemic accentuated telework. However, the question of “who is engaged in remote work” remains unanswered [24] and seems to be accentuated after the outbreak of COVID-19. Without trying to answer this question, in our research all respondents were employed. Studies have been conducted to predict what will happen in the telecom industry during and after the COVID-19 pandemic [25,26,27,28,29]. Accordingly, the social elements of employees should be taken into consideration to increase the number of teleworkers available. One of them would be to raise awareness among employees about the advantages of teleworking, such as the decrease in commute time, especially when traveling large distances. According to research [30], it might be a practical solution to transportation-specific issues such as traffic congestion, but also to filthy/polluted air in metropolitan areas in developing nations. 

A deciding factor in telework is also the age of the workers [31,32,33,34,35,36]. Thus, the theoretical arguments identified in some research support the idea that human resource practices, such as teleworking, flexible working hours or work-life balance, could be effective in discouraging older workers that may be replaced by young people to leave an organization voluntarily [37]. The COVID-19 pandemic experience has been the subject of an increasing number of recent studies, which aim, among other things, to investigate how the subjective well-being of workers is influenced and influences labor productivity in the context of a large-scale implementation of teleworking solutions [38]. The findings of this study may come as a surprise in that the effects on the ability to work from home have been reported more frequently due to indoor noise sources, particularly airborne noise and impact noise from neighboring suites, rather than from outdoor noise sources.

## 3. Research Strategy

The objective of this research is to identify the economic and social impact of telework in changing the behavior of employees in Romania. An analysis of literature highlighted the evolution of remote work culminating in the emergence of telework since the proliferation of information and communication technologies. The COVID-19 pandemic accentuated the need for telework and imposed major changes in the approach of remote activities. Thus, new regulations and norms that led to the completion of labor legislation emerged. The research was conducted approximately one year after the onset of the pandemic until the beginning of the vaccination period in Romania.

To obtain conclusive results, we used a mixed methodology, combining results obtained through a survey based on a self-administered electronic questionnaire, with a documentation analysis and a data mining analysis. Detailed analysis of the groups identified based on work efficiency allowed us to highlight the most common employee profiles. This analysis was doubled by a second classification experiment, which provided us a more detailed analysis of the groups identified based on job satisfaction and highlighted the most common employee profiles.

The literature highlights a number of models from the models of conventional prediction methods using decision trees, the ARIMA prediction model. K- Nearest Neighbors, cluster models, multivariate linear regression prediction models [39].

The proposed research includes three main directions of analysis of the data extracted, which are related to the efficiency of work during telework, this being considered one of the most important indicators for the company. To obtain conclusive results, we used a mixed methodology, combining the results obtained through a survey based on a self-administered electronic questionnaire with a documentation analysis and a data mining analysis. Detailed analysis of the groups identified based on work efficiency allowed us to highlight the most common employee profiles. This analysis was doubled by a second classification experiment that provided us with a more detailed analysis of the groups identified based on job satisfaction and highlighted the most common employee profiles.

To highlight the gaps encountered in the literature in the documentary analysis, we used the opinion poll based on the electronic questionnaire type CAWI (Computer Assisted Web Interviewing/Online Research). The CAWI system is a fast method of collecting survey data, based on a standardized questionnaire via the Internet. The specialized software of CAWI ensured the editing and management of the questionnaire; generated the sample based on a sampling frame represented by a database with e-mail addresses; eliminated duplication of respondents by generating a unique identification code for each email address.

Based on the objective of the research, meaning to identify the economic and social impact of telework in changing the behavior of employees in Romania, the proposed research contains three main directions of the data mining analysis which are related to the work efficiency during teleworking (TL) (1), work satisfaction felt by the employees during TL (2) and perhaps to the factors that have the greatest impact on the employee in terms of changing the mentality regarding work in telework and its use after the disappearance of the COVID-19 pandemic (3) (Table 1). The (1) and (2) where analyzed based on the economic and social factors from the employee point of view. The main analysis directions that are conducted for this study try to respond to the following research questions:

Q1: which are the most important economic and social factors that influence the work efficiency of TL versus normal way of working and which are the main characteristics of the employee groups that feel different regarding the work efficiency during TL?

Q2: which are the most important economic and social factors that influence the work satisfaction during TL and which are the main characteristics of the employee groups that feel different regarding the work satisfaction during TL?

Q3: which are the main characteristics of the employee groups that feel different regarding the impact of TL after the pandemic will pass? 

Q4: which are the main profiles for the employees regarding the work efficiency during TL?

Q5: which are the main profiles for the employees regarding the work satisfaction during TL? 

In Q1 and Q2 we aimed to identify the economic and social factors that influence efficiency and job satisfaction (we applied clustering algorithms). In Q4, Q5 we want to identify customer profiles for efficiency and job satisfaction (based on clustering data and we have applied classification algorithms to identify detailed information at the customer profile level).

Table 1 shows the directions of analysis taking into account the factors that influence the efficiency and satisfaction felt during teleworking. We also considered the analysis of the factors that can change the mentality regarding the use of telework, taking into account the satisfaction and efficiency felt in telework. The elements are correlated with the 5 research questions mentioned above. 

We mention that all the elements from Table 1, respectively the attribute groups and the attribute classes are explained in Table A1 in the annex.

## 4. Research Methodology

**Figure A1:**
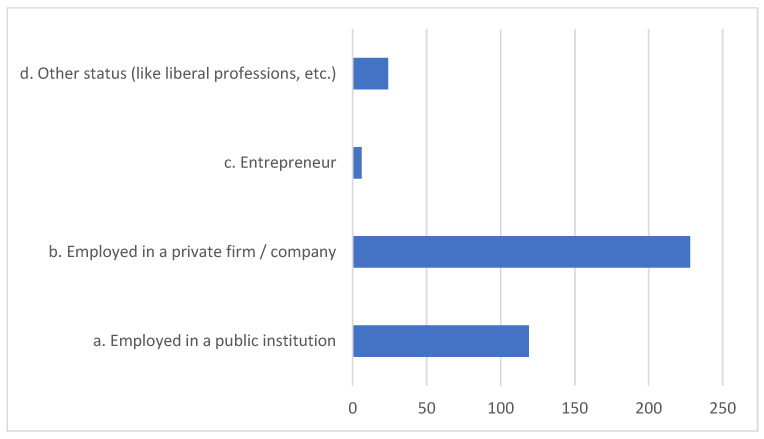
Status of respondents on the labor market.

**Figure A2:**
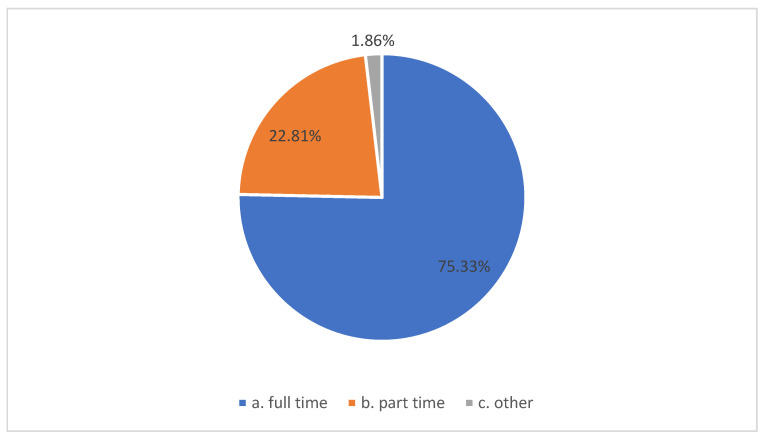
Part-time or full-time employees.

**Figure A3:**
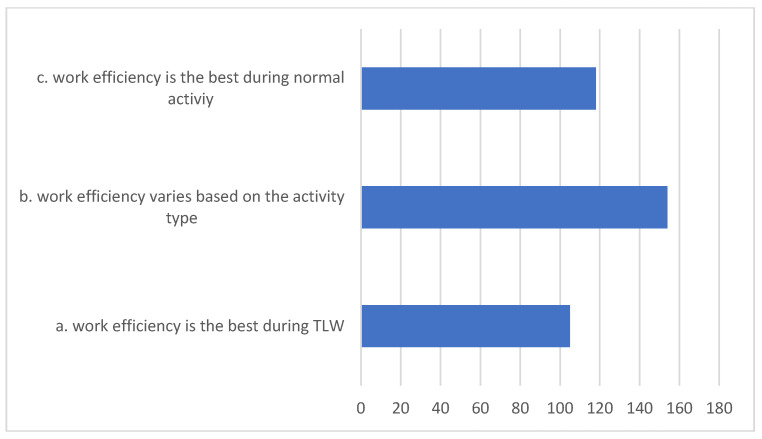
Efficiency of teleworking activity vs. office efficiency.

**Figure A4:**
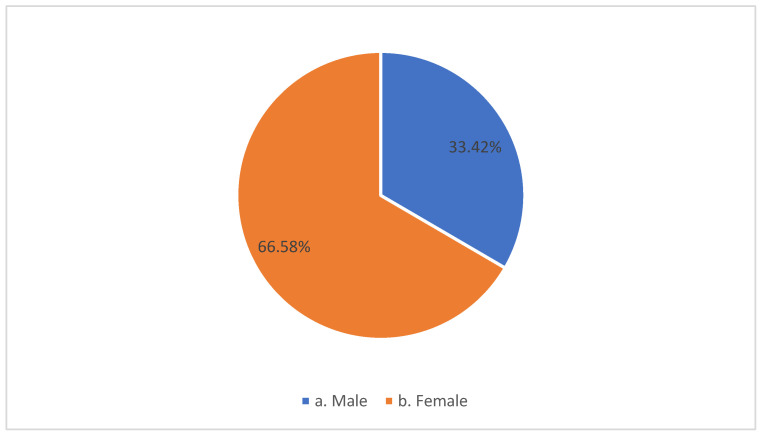
Gender of respondents.

The research is made using data collected from employees that are working for state and private companies. In order to collect data, an online survey was created that was sent to over 450 employees. The questionnaire was completed by 450 respondents. After verifying them, we found that only 377 questionnaires contained sufficient information for analysis, the others being partially completed. In the end, after the data pre-processing, 377 valid records remained to be analyzed). The questionnaire is available in the Appendix A among with the statistics (Figure A1, Figure A2, Figure A3 and Figure A4). The survey was structured in several parts as follow:

- questions regarding personal and professional information of the employee;
- questions about home accommodation of the employee for TL;
- the support that the employer has offered to the employee during TL;
- impact of TL over the employee regarding social and professional aspects;
- factors that may change the point of view over the TL after the pandemic will pass;
- work efficiency and satisfaction during TL;
- economic consequences of TL;

Data analysis was made using a specific approach of data mining methodology. For this research it was used the DM–CRISP methodology (https://www.datascience-pm.com/crisp-dm-2/, accessed on 28 June 2021) which consists in several steps, namely: requirements understanding, data understanding, data preparation, data modeling, evaluation, and deployment. We undermine the correlation that could be created between economic and social factors in terms of efficiency and job satisfaction.

The data mining dataset obtained at the end of the data preparation step contains 41 attributes and 377 records. The main activities made over the dataset were:

- collecting the data from the questionnaire that was sent to employees in order to be filled in with their opinion about TL. In this regard, economic and social information was collected.
- establishing the degree of filling for each attribute and eliminating the empty records or with very few data; By degree of filling is meant how many values an attribute had completed.
- the excel file with the preprocessed data was transformed into an arff type file that was used in the Weka data mining software for the analysis [40,41].

The attributes were divided in several groups. These groups are presented in Table A1 from the Appendix A. The reason of this division was to have an approach from economic and social perspectives of TL, from the employee’s point of view. The main groups are the following, for each one being mentioned the comprised information:

- Group A (social)—personal information about the employee, information about last level of studies, geographic region from Romania where he/she lives, age, and sex;
- Group B (social)—information about the employee’s professional activity such as status on labor market, if he/she is employed or not, if he/she performed TL in 2020, salary level, activity domain;
- Group C (economic)—information on home accommodation for teleworking, if any additional investments were made for TL, if any additional expenses were made for TL regarding services like electricity, telephony, internet, water and sanitation, heating and air conditioning and also their total estimation, how much the respondent was informed by the government about the TL regulations;
- Group D—support provided by the employer for teleworking with information about the aspect that identifies if the employer made efforts to facilitate work during TL and if he/she has offered continuously support for employees;
- Group E (economic)—efficiency of teleworking vs. normal activity with information about how the employee has considered the efficiency of the TL over the normal activity;
- Group F—information about the employer (company) regarding the number of employees and if the regulations given by the state had impact on supporting the TL for the employees;
- Group G (economic)—employee and TL in relation to basic activities with information about how the TL is appropriate for the employee to carry out main activities;
- Group H (economic)—consequences of teleworking with information about the additional hours allocated for TL and the variation of the income;
- Group I (social)—the impact of teleworking from a social point of view with information about communication efficiency during TL, main advantages and disadvantages of TL, main problems identified by the employees;
- Group J—factors ranking with information about several types of factors (like cultural, economic, natural, psychological, social) that are most affected by the TL;
- Group K (social)—satisfaction degree with information about if the employee felt satisfaction during TL;
- Group L—impact of TL after the pandemic with information about the fact if the employee is willing to continue in TL after the pandemic is gone or he/she wants to return to the office for full time;

The most relevant economic factors that have an impact over the employee are comprised in group B and the most relevant social factors are in group I. The other groups completed the analysis with other information regarding the teleworking.

The algorithms that were used for the data mining analysis:

- establishing the degree of filling for each attribute;
- ranking the attributes related to a specific class attribute: *GainRatioAttributeEval* algorithm as attribute evaluator was used together with *Ranker* search methods; a ranking score was obtained for each attribute used, based on a class attribute; the scores are offered in a decreasing order and the attribute with the greatest score is the most important (or relevant) to the class attribute; Some algorithms (e.g. “GainRatioAttributeEval”, “PART”, “J48”) are required to obtain intermediate results. They were mentioned to make the analysis more transparent.
- cluster algorithms: Simple K-Means to determine the characteristics for each group for a certain number of employees group. Before using the Simple K-Means algorithm, the EM (expectation maximization) algorithm was used in order to identify the most appropriate number of groups (clusters); The EM algorithm was used to identify the number of clusters in the dataset. Then the Simple K-Means algorithm was used for cluster analysis.
- classification algorithms: The PART algorithm for generating a decision rules list and the J48 algorithm to obtain profiles for employees using classification decisions trees. Both algorithms are related to a certain class attribute.

## 5. The Modeling Phase and the Main Results

Two types of data mining approaches are used to analyze the dataset collected based on questionnaire (Table 2). The first one is based on clustering approach for which the relevance of the prediction variables is determined. The second data mining approach is based on classification trees. The reasons the classification trees are a better option over the traditional statistical models (like for example discriminant analysis) are because they can deal with a high number of predictor variables, and they can highlight non-linear relationships and complex interactions between dependent variables and predictors. Both analyses were made using the Weka data mining platform (https://www.cs.waikato.ac.nz/mL/weka/, accessed on 28 June 2021).

### 5.1. Clustering Approach

#### 5.1.1. First Cluster Experiment

The first cluster experiment tries to respond to the Q1 research question. A number of 23 attributes out of 41 are selected in order to identify the relationship between the work efficiency that employees felt that they had while they were in TL way and certain social and economic factors. The number of clusters was obtained by using the K-Means algorithm and the parameter that calculated the distance used the Euclidean distance. The factors used are from groups A (Personal information about the employee), B (Information about the employee’s professional activity), C (Home accommodation for teleworking), D (Support provided by the employer for teleworking), F (Information about the employer/company), G (Employee—telework in relation to basic activities). Based on these factors a ranking scale is determined and the most important factors related to the efficiency during TL were identified. For this analysis, two algorithms were used together, namely the *Gain Ration Attribute Evaluator* which evaluates the worth of an attribute by measuring the gain ratio with respect to a class attribute and *Ranker Search Method* which ranks attributes by their individual evaluations. The class attribute for this experiment is the one for which the employee has mentioned the work efficiency level during TL over the normal activity (Efficiency TLW vs. normal activity attribute, see Appendix A, Table A1). Applying these two algorithms, each attribute receives a ranking score based on the used class attribute. The ranking scores are offered in a decreasing order. The attribute with the greatest score is the one that is the most important for the class attribute and the attribute with the lowest score is the less important for the class attribute. The results for the most important ten factors that are relevant for the TL work efficiency are mentioned below. For example, the first attribute, which represents the employee’s opinion about TL being appropriate to do the main job activities, has received the biggest score equal to 0.150244. 

*0.150244 TLW is appropriate in doing the main activities (group G)*

*0.130096 Home accommodation for TLW (group C)*

*0.070768 Employer effort to facilitate TLW (group D)*

*0.056325 Regulations impact supporting TLW on your employer (group F)*

*0.047409 Employer continuously support for TLW (group D)*

*0.031126 Last level of studies (group A)*

*0.026247 Information degree about government regulations on TLW (group C)*

*0.022289 Geographic region (group A)*

*0.01736 Amount of the additional investments for TLW (group C)*

*0.017109 Additional expenses for TLW (group C)*

Taking into account the results of this ranking scale we can say that the most important five factors that are influencing the work efficiency of the employees during TL depend very much on:

- the job particularities and its activities and how well these can be carried out in a TL way;
- how much the home of the employee can be transformed into a working place or office and the accommodation degree;
- the employer and how much effort it puts into facilitating the working activities of the employee;
- the state and the laws that it gives in order to help and sustain the employer during this TL period of time;
- the implication of the employer in offering a continuously support to the employers during the hole teleworking period.

Furthermore, a clustering model is developed in order to identify the characteristics of each group of employees depending on the efficiency degree that they felt during teleworking. For this model 23 factors were used also, the same that were ranked above. After using the EM algorithm, three clusters were indicated. For this number of clusters, the K-Means algorithm was applied. The results offered by the K-Means algorithm for the three clusters consist in Cluster 0 (165 records which represents 44% from the all-dataset), Cluster 1 (127 records which represents 34% from the all-dataset) and Cluster 2 (85 records which represents 23% from the all-dataset). The details for each cluster are mentioned in Table 3. 

The characteristics of the employees related to the social and economic factors that were analyzed and considering the work efficiency is described based on the most five important factors, according to the ranking scale. The employees that are represented by the Cluster 0 and considered that they have the best efficiency in the teleworking way, according to the cluster centroid (the most representative record from the group):

- have graduated a faculty as last level of study.
- are between 26 and 40 years old.
- have a salary amount between 2501 and 4000 RON (equivalent of 500 and 800 Euro);
- they work in the economic domain in a company that has between 50 and 250 employees;
- have made a lot of initial investments between 501 and 1000 RON (equivalent of 100 and 200 Euro) for TL home accommodation;
- have had additional expenses per month between 100 and 500 RON (equivalent of 20 and 100 Euro), this being for electricity, telephony, internet, water and sanitation, heating and air condition;

They also consider that:

- home accommodation was done properly;
- the employer made effort to facilitate the teleworking and to offer continuously support in order to sustain it;
- the regulations offered by the state have a high impact in helping the employers.

The employees that are represented by Cluster 1 and who considered the efficiency varies based on the activities in the TL way:

- have graduated a high school as last level of study;
- are between 18 and 26 years old;
- have a salary amount between 1300 and 2500 RON (equivalent of 260 and 500 Euro);
- they work in the economic domain in a company that has between 10 and 49 employees;
- have not made any initial investments for TL home accommodation
- did not have any additional expenses or very few per month, under 100 RON (equivalent of 20 Euro), this being for electricity, water and sanitation, heating and air condition;

They also consider that:

- home accommodation was done properly;
- the employer made effort to facilitate the teleworking and to offer continuously support in order to sustain it;
- the regulations offered by the state have a high impact in helping the employers.

The employees that are represented by Cluster 2 and who considered the work efficiency is not good at all and the normal activity way is the best:

- have graduated a faculty as last level of study;
- are between 40 and 55 years old;
- have a salary amount between 2501 and 4000 RON (equivalent of 500 and 800 Euro);
- they work in services domain in a company that has between 10 and 49 employees;
- have not made any initial investments for teleworking home accommodation;
- did not have any additional expenses or very few per month, under 100 RON (equivalent of 20 Euro), this being only for electricity;

They also consider that:

- home accommodation was not done properly;
- the employer made no effort to facilitate the teleworking and to offer continuously support in order to sustain it;
- the regulations offered by the state have a high impact in helping the employers.

The three clusters are different based on several characteristics like age and experience, money that the employees have spent for home accommodation and expenses, salary level point of view and from the support offered by the employer, last level of the studies. All of them agree that the regulations offered by the state have a high impact in helping the employers’ activity.

#### 5.1.2. Second Cluster Experiment

The second cluster experiment tries to respond to the Q2 research question. A number of 8 attributes out of 41 are selected in order to identify the relationship between the work satisfaction degree that employees felt during TL and certain social and economic factors. The factors used are from groups H (Consequences of teleworking), I (The impact of teleworking) and E (Efficiency of teleworking vs. normal activity). Based on these factors a ranking scale is determined and the most important factors related to the work satisfaction during teleworking were identified. For this analysis the *Gain Ration Attribute Evaluator* was used among with the *Ranker Search Method*. The class attribute for this experiment is the one for which the employee has mentioned the work satisfaction level during TL (11_K_Satisfaction_degree_for_TLW_activity attribute from Appendix A, Table A1). The most important ten factors for the work satisfaction felt by the employees while they were in TL are mentioned below. 

*0.10169 Efficiency TLW vs. normal activity (Group E)*

*0.07404 TLW positive influence over the communication efficiency (Group I)*

*0.03809 Income level during the pandemic (Group H)*

*0.01805 Disadvantages of TLW (Group I)*

*0.01602 Main disadvantage of TLW (Group I)*

*0.0147 Problem of TLW (Group I)*

*0.01297 TLW means also additional working hours (Group H)*

*0.00712 Main advantage of TLW (Group I)*

Considering the results of this ranking scale we can say that the most important five factors that are influencing the work satisfaction of the employees during TL depend very much on:

- the efficiency that the employee felt he had during the TL;
- if the employee felt that the communication efficiency was influenced by the TL;
- the income level during the pandemic being in TL;
- the employee identifies disadvantages and problems for teleworking;
- if the employee must work additional hours.

A clustering model is developed in order to identify the characteristics of each group of employees depending on the work satisfaction felt during teleworking. For this model also 8 factors were used, the same that were ranked above for the second cluster experiment. After using the EM algorithm, three clusters were indicated. For this number of clusters, the K-Means algorithm was applied. The results offered by the K-Means algorithm for the three clusters consists in Cluster 0 (100 records which represents 27% from the all-dataset), Cluster 1 (149 records which represents 40% from the all-dataset) and Cluster 2 (128 records which represents 34% from the all-dataset). The details for each cluster are mentioned in Table 4.

The characteristics of the employees related to the social and economic factors that were analyzed and taking into account the efficiency that is described based on the most five important factors, according to the ranking scale. The cluster analysis results are presented in Table 4 among with the values for all the factors. 

The employees represented by Cluster 0 have considered they felt a high work satisfaction degree for TL, according to the cluster centroid:

- the work efficiency is best in TL versus normal working way;
- teleworking does not imply additional working hours;
- the communication efficiency was improved by the teleworking;
- the work satisfaction was high during teleworking;
- the main advantage is considered to be the time saved that was spent in traffic;
- the main disadvantage of TL is considered to be the lack of human interaction;
- the communication level has decreased between team members and this it is considered to be an important problem in order to develop daily activities and tasks;
- the income increased during the TL.

The employees represented by Cluster 1 have considered they felt an average work satisfaction degree for Teleworking, according to the cluster centroid:

- the work efficiency varies based on the activity type;
- teleworking does imply additional working hours;
- the communication efficiency was not improved by the teleworking;
- the work satisfaction was average during teleworking;
- the main advantage is considered to be the time saved that was spent in traffic;
- the main disadvantage of teleworking is considered to be the lack of human interaction;
- the lack of the direct contact between employees and customers it is considered to be an important problem;
- the income remained the same during the teleworking.

The employees represented by Cluster 2 have considered they felt an average work satisfaction degree for Teleworking, according to the cluster centroid:

- the income remained the same during the teleworking.
- the work efficiency is best during the normal working way;
- teleworking does not imply additional working hours;
- the communication efficiency was not improved by the teleworking;
- the work satisfaction was average during teleworking;
- the main advantage is considered to be the time saved that was spent in traffic;
- the main disadvantage of teleworking is considered to be the lack of human interaction;
- the communication level has decreased between team members and this it is considered to be an important problem in order to develop daily activities and tasks;
- the income remained the same during the teleworking.

The main differences between the three clusters are for the work satisfaction degree during teleworking, the efficiency, income level, the additional working hours and the income salary. It can be concluded that the work satisfaction is much related to the efficiency and the information for these clusters can be completed with the information from the first cluster analyses based on the values of the work efficiency factor. 

#### 5.1.3. Third Cluster Experiment

The third cluster experiment tries to respond to the Q3 research question. A number of 10 attributes out of 41 are selected in order to identify the relationship between the impact of teleworking after the pandemic (group L) and several factors like the ones from groups B (activity domain), K (work satisfaction), E (work efficiency), J (different factors ranked by respondents, factors like economic, social, cultural, psychological and natural) and I (main advantage and main disadvantage). For this analysis the *Gain Ration Attribute Evaluator* was used among with the *Ranker Search Method*. The class attribute for this experiment is the one for which the employee has mentioned his will to return to the office or to stay in TL after the pandemic is gone (20_L_TLW_after_the_pandemic attribute from Appendix A, Table A1). The most important ten factors that affect the decision of the employee to remain in TL or not are mentioned below.

*0.1848 Satisfaction degree for TLW activity (Group K)*

*0.13529 Efficiency TLW vs. normal activity (Group E)*

*0.05557 Activity domain(Group B)*

*0.02656 Factors influence POV about TLW 5th place (Group J)*

*0.01597 Main advantage of TLW(Group I)*

*0.01578 Factors influence POV about TLW 4th place (Group J)*

*0.01475 Factors influence POV about TLW 1st place (Group J)*

*0.01394 Main disadvantage of TLW (Group I)*

*0.00617 Factors influence POV about TLW 3rd place (Group J)*

*0.00561 Factors influence POV about TLW 2nd place (Group J)*

A clustering model is developed in order to identify the characteristics of each group of employees depending on the will to remain or not in TL after the pandemic. For this model also 10 factors were used, the same that were ranked above for the third cluster experiment. Using the Simple K-Means algorithm, three clusters are determined, namely: Cluster 0 (142 records which represents 38% from the all-dataset), Cluster 1 (144 records which represent 38% from the all-dataset) and Cluster 2 (91 records which represent 24% from the all-dataset). The details for each cluster are mentioned in Table 5.

The employees represented by the profile of Cluster 0 consider TL the best way for working and they will want to continue working in this way after de pandemic will pass:

- the work efficiency is best during teleworking;
- the work satisfaction is average;
- main advantage is the saved time that was spent in traffic;
- main disadvantage is the lack of human interaction;
- the order of the factors that can influence the point of view over the teleworking is: economic, social, psychological, cultural and natural;
- activity domain is in Services.

The employees represented by the profile of Cluster 1 consider that the TL is good only for certain type of activities and will want to continue only for these after the pandemic will pass:

- the work efficiency is good only for certain activities;
- the work satisfaction is average;
- main advantage is the saved time that was spent in traffic;
- main disadvantage is the lack of human interaction;
- the order of the factors that can influence the point of view over the teleworking is: social, economic, psychological, cultural and natural;
- activity domain is in Economic.

The employees represented by the profile of Cluster 2 consider that the TL is no good at all and after the pandemic passes, they will want to come back to the office:

- the work efficiency is no good at all in TL and they want to come back to the normal working way as soon as possible;
- the work satisfaction is average in TL;
- main advantage is the saved time that was spent in traffic;
- main disadvantage is the lack of human interaction;
- the order of the factors that can influence the point of view over the TL is: psychological, social, economic, natural and cultural;
- activity domain is Economic.

### 5.2. Classification Approach

The classification approach is realized using two algorithms. The first one is PART algorithm which offers as a result a decision rules list that contains the most encountered rules that characterized the responses for the employees from the dataset. The second one is J48 algorithm which offers as a result a decision tree that classify the instances based on a specific attribute (class attribute).

#### 5.2.1. First Classification Experiment

The first classification experiment tries to respond to the Q4 research question. The objective of this experiment is to offer a more detailed analysis of the clusters identified based on the work efficiency in the first cluster experiment and to highlight the most common profiles of the employees. There are taking into account social and economic factors that were used also for the first cluster experiment. The attributes used are from groups regarding personal information (group A), employee’s professional activity (group B), home accommodation for TL (group C), the support provided by the employer for TL (group D), the employer (group F) and the employee and the relation between TL and basic activities (group G). 

Using the PART algorithm, a list that contains 22 decision rules is obtained. Some of the most representative decision rules for each work efficiency-based cluster are:

*First rule*

*Additional expenses for TLW = Yes quite a lot (group C) AND*

*Additional investments for TLW = Yes (group C) AND*

*Additional expenses INTERNET = Yes (group C): cluster 0 (114.0/3.0)*

First rule comment: the employees for whom this rule can be applied, during teleworking had a lot of additional expenses during this period and initial investments starting the teleworking. The internet has also represented an important expense. For these employees the work efficiency was considered to be the best during teleworking over the normal working way (Cluster 0 from the first cluster experiment which has contained 44% from the all-dataset). This rule is matching to 114 instances from the dataset.

*Second rule*

*Additional expenses for TLW = Not at all or very few (group C) AND*

*Employer continuously support for TLW = Not sustained (group D) AND*

*TLW appropriate in doing the main activities (group G) = No AND*

*Age = between 40 and 55 (group A): cluster 2 (15.0)*

Second rule comment: the employees for whom this rule can be applied are between 40 and 55 years old, during teleworking did not have additional expenses during this period or very few and they have not received continuously support from the employer. They also consider that their main activities are not suitable for teleworking way. For these employees the work efficiency was considered to be not good at all while and they prefer the normal working way (Cluster 2 from the first cluster experiment which has contained 22% from the all-dataset). This rule is matching to 15 instances from the dataset.

*Third rule*

*Additional expenses for TLW = Not at all or very few (group C) AND*

*Employer continuously support for TLW = Sustained (group D) AND*

*Additional investments for TLW = No (group C) AND*

*Additional expenses WATER and SANITATION = Yes (groupC): cluster 1 (45.0/1.0)*

The third rule comment: The employees for whom this rule can be applied, during teleworking did not have additional expenses during this period or very few, they have not made any additional investment and have received continuously support from the employer. They also consider that the expenses for water and sanitation have increased. For these employees the work efficiency was considered to vary based on the activity characteristics (Cluster 1 from the first cluster experiment which has contained 34% from the all-dataset). This rule is matching to 45 instances from the dataset.

Using the J48 algorithm, a decision tree is obtained which has the size equal to 67 and the number of leaves equal to 47. For this decision tree rules from the PART algorithm can be identified but much more cases are described.

The characteristics for the results are: Correctly Classified Instances: 302 meaning 80.1%, Incorrectly Classified Instances: 75 meaning 19.9%, Kappa statistic (means “fulfill the prediction level”, this value is considered to be a good one, maximum being 1): 0.6892, Mean absolute error: 0.1637, Root mean squared error: 0.3451, Relative absolute error: 38.1074%, Root relative squared error: 74.4639%, Total Number of Instances: 377.

The confusion matrix (Table 6) shows that for the first line 150 instances were correct classified into Cluster 0 (a), 11 instances were incorrect classified into Cluster 1 (b) and they should have been classified in Cluster 0 and 4 instances were incorrect classified into Cluster 2 (c) and they should have been classified in Cluster 0.

Based on the confusion matrix, indicators like TP rate (True-Positive rate), Recall, Precision, F-Measure (computed as 2*Precision*Recall/(Precision +Recall). The values regarding the accuracy of the results by class attribute for this experiment are (Table 7):

A part of this decision tree is mentioned below. For this part of the tree, we can say that for the first three conditions 114 instances are correct classified and the attribute related to the additional expenses for teleworking (08_C_Additional_expenses_for_TLW attribute from the Appendix A, Table A1) has a big influence on the work efficiency. These 114 instances are classified into Cluster 0 for which the work efficiency was the best during teleworking.

*Additional expenses for TLW = Yes_quite_a_lot (group C)*

*| Additional investments for TLW = Yes (group C)*

*| | Additional expenses INTERNET = Yes (group C): cluster 0 (114.0/3.0)*

Another part of the decisions tree says that a common profile of an employee is characterized by the fact he had a lot of additional expenses, additional investment in order to be able to start working from home, the expenses with the internet were not a big problem and the region from Romania where he lives is Muntenia South (Sud_Muntenia). This profile is common to cluster 1 in which the work efficiency is considered to vary based on the activity characteristics.

*Additional expenses for TLW (group C) = Yes quite a lot*

*| Additional investments for TLW (group C) = Yes*

*| | Additional expenses INTERNET (group C) = No*

*| | | Geographic_region = Sud_Muntenia (group A): cluster1 (10.0/3.0)*

#### 5.2.2. Second Classification Experiment

The second classification experiment tries to respond to the Q5 research question. The objective of this experiment is to offer a more detailed analysis of the clusters identified based on the work satisfaction in the second cluster experiment and to highlight the most common profiles of the employees. There are taking into account social and economic factors that were used also for the second cluster experiment. The attributes used are from groups H, I and E.

Using the PART algorithm, a list that contains 21 decision rules is obtained.

Some of the most representative decision rules for each work satisfaction-based cluster are:

*First rule*

*Efficiency TLW* vs. *normal activity = NormActiv_best (group E) AND*

*TLW means also additional working hours = No (group H) AND*

*TLW positive influence over the communication efficiency = No (group I): cluster 2 (64.0)*

First rule comment: the employees for whom this rule can be applied consider that the normal working way is the best; they did not take additional working hours and consider also that the teleworking did not have a good influence over the communication. For these employees the work satisfaction was considered to be average during teleworking (Cluster 1 from the second cluster experiment which has contained 34% from the all-dataset). This rule is matching to 128 instances from the dataset.

*Second rule*

*TLW positive influence over the communication efficiency = No (group I) AND*

*Satisfaction degree for TLW activity = Average (group K)AND*

*TLW means also additional working hours = Yes (group H)AND*

*Efficiency TLW* vs. *normal activity = Varies based on activity (group E): cluster 1 (35.0)*

Second rule comment: the employees for whom this rule can be applied consider that teleworking did not have a good influence over the communication, they did additional working hours and the work efficiency depends on the activity type. For these employees the work satisfaction was considered to be average during teleworking (Cluster 1 from the second cluster experiment which has contained 40% from the all-dataset). This rule is matching to 149 instances from the dataset. 

*Third rule*

*Efficiency TLW* vs. *normal activity = TLW best (group E) AND*

*TLW positive influence over the communication efficiency = Yes (group I) AND*

*TLW means also additional working hours = No (group H): cluster 0 (36.0)*

Third rule comment: the employees for whom this rule can be applied consider that work efficiency during teleworking was the best over the normal working way, teleworking did have a good influence over the communication and no additional working hours were made. For these employees the work satisfaction was considered to be high during teleworking (Cluster 0 from the second cluster experiment which has contained 27% from the all-dataset). This rule is matching to 100 instances from the dataset. 

Using the J48 algorithm, a decision tree is obtained which has the size equal to 56 and the number of leaves equal to 46. 

The characteristics for the results are: Correctly Classified Instances: 343 meaning 91%, Incorrectly Classified Instances: 34 meaning 9%, Kappa statistic: 0.8627, Mean absolute error: 0.0791, Root mean squared error: 0.2293, Relative absolute error: 18.0326%, Root relative squared error: 48.9588%, Total Number of Instances: 377. 

The confusion matrix (Table 8) shows large numbers on the main diagonal which means many instances were correctly classified. This fact is correlated to the 91% of the correctly classified instances mention above.

Based on the confusion matrix, the following indicators are computed regarding the accuracy of the results by class attribute (Table 9).

A part of this decision tree is mentioned below. 

*Efficiency TLW* vs. *normal activity = TLW best (group E)*

*| TLW positive influence over the communication efficiency = Yes (group I): cluster 0 (58.0/2.0)*

For this part of the tree, we can say that the work efficiency is the best during TL and also the communication is more efficient being influenced in a positive way by this type of working. These 58 instances are classified into Cluster 0 for which the work satisfaction was high during teleworking.

Another part of the decision tree described below is presenting a profile for the Cluster 2 from the second cluster experiment. For this profile the work efficiency varies based on the activity type, no additional working hours are made, the main identified problem of teleworking is that the communication level between team members is decreasing and there is no positive influence over the communication from the teleworking way. These 18 instances are classified into Cluster 2 for which the work satisfaction was average during teleworking.

*Efficiency TLW* vs. *normal activity = Varies based on activity (group E)*

*| TLW means also additional working hours = No (group H)*

*| | Problem of TLW = Decreasing the communication level between team members (group I)*

*| | | TLW positive influence over the communication efficiency = No (group I): cluster 2 (18.0)*

## 6. Findings and Discussion

Recent studies consider that the implementation of remote work is generally beyond a person’s control. It requires some form of social support. For the promotion of distance-based work, it is considered necessary to change the paradigm, at the level of organizational management, communication methods and workflow processes [42]. Researchers point out that the best teleworkers pay special attention to the effective management of the border between work and home, discipline being essential [43,44].

In the previous section, several experiments were conducted in order to obtain the answers to the main research questions. In this section the main findings are mentioned—that is, from the social and economic points of view. 

Q1: which are the most important economic and social factors that influence the work efficiency of TL versus normal way of working and which are the main characteristics of the employee groups that feel different regarding the work efficiency during TL?

The most important economic and social factors that influence the work efficiency of TL are regarding: the job type and its particularities, how well the activities may be accomplished during TL, the flexibility of the employee to transform his/her home or part of his/her home into an office and not to be disturbed, the involvement of the employer in facilitating the TL for the employee, the involvement of the state based on the laws and regulations that it creates in order to sustain and to create a framework more appropriate for the employer during the TL, the continuously support that the employer is offering to the employee during the entire TL period of time.

As it can be observed, the work efficiency of the employee depends very much on all actors that are involved, actors like employer, state, and employee. Each one can, in one way or another, improve the work efficiency. However, finding the best solutions and, in some cases in a very short period, is not an easy thing to accomplish. For this reason, all the actors must collaborate to identify them to achieve the best work efficiency during TL. 

Regarding the groups of employees obtained in relation with the work efficiency, we may conclude the following (only two groups were described, one with the best work efficiency and another one that did not have a good work efficiency):

- the employees that have considered having the best work efficiency: they work in the economic domain, in which many activities may be carried out in TL; they made additional investments to organize a space from home into an office (between 100 and 200 Euro) and had monthly additional expenses (between 20 and 100 Euro). They also received support from the employer in facilitating TL and from the state based on the adopted regulations. So, for this situation, all the three actors made efforts to improve the work efficiency—the employee made investments to transform his/her home, the employer offered support and the state has created the appropriate legal framework for TL.
- the employees that have considered not having a good work efficiency at all and wanted to come back to the office work in the Services domain, in which only some activities may be performed in a proper manner in TL; they have not made any additional investments for home accommodation and the monthly additional expenses are low (under 20 Euro). In this situation, even if they consider that the state has created a good legal framework for TL, they consider also that the employer made no effort to help them and also they were not open to transform the home into an office and to create the right accommodation.

**Implications of TL in terms of work efficiency.** As it can be seen, the work efficiency during TL depends very much on the activity domain and its characteristics. For this reason, each domain should be analyzed and a legal framework for TL should be created, considering its particularities. Thus, the work efficiency in TL may be increased also in the work sectors in which the TL activity is not very suitable. However, the design of such a legal framework that takes into account each domain is a complex thing to do but, such a framework can, in some situations, further force the support provided by employers. In this situation, it is expected that it will be easier for employees to get involved in the TL.

Q2: which are the most important economic and social factors that influence the work satisfaction during TL and which are the main characteristics of the employee groups that feel different regarding the work satisfaction during TL?

The most important economic and social factors that influence the work satisfaction of TL are regarding first of all the work efficiency during TL, then the communication efficiency and on the third place the income that for many employees has encountered big variations. For example, in Romania, many employees have received during TL an income reduced with 25%. However, all the employees have received an income during TL. In some cases, also the additional working hours was a factor that has influenced the work satisfaction during TL. 

Regarding the groups of employees obtained in relation with the work satisfaction, we may conclude the following (only two groups described, one with the highest work satisfaction and another one that had an average work satisfaction):

- the employees that have considered having the highest work satisfaction: have the best work efficiency in TL, are able to communicate in an efficient manner with their colleagues and the distance in not a problem to them and, also their income has increased and no additional hours were made. So, in this category we may conclude that there are employees that were able to adapt quickly, to understand the situation and to overcome easily the challenges imposed by the TL. However, we may also say that their domain was not affected too much by the pandemic or it was developed even more (like the IT domain).
- the employees that have considered having an average work satisfaction and wanted to return to the office as soon as possible: they consider the office work the best manner to do the job and to accomplish the activities, the TL had a negative effect for the communication between colleagues, and the income remained the same.

For both groups described, the main advantage is the time saved from staying in traffic and the main disadvantage is the lack of human interaction. 

Implications of TL in terms of work satisfaction. The work satisfaction, as the analysis has showed, is much related to the work efficiency and to the communication efficiency between colleagues. For these reasons, it can be said that it is also related to the activity domain, but also to the context that each employee has. However, it is well known that the work satisfaction in general is a sum of several elements. These elements, beside work efficiency and colleagues, we may consider to be: income, supervision (the degree to which superiors demonstrate consideration and interest for employees), and promotion. Therefore, in order to improve the work satisfaction, the employers may play an important role in increasing it. Employers may think and try new ways for the employees to communicate between them, to supervise them in a more efficient manner, to offer them feedback more rapidly and in a more professional way. The employers may also let the employees know that even during the pandemic a promotion might be possible and also an increased salary may be offered. 

Q3: which are the main characteristics of the employee groups that feel different regarding the impact of TL after the pandemic will pass? 

The main characteristics of the employee groups that feel different regarding the impact TL after the pandemic are as following (only two groups were described, one that will want to work only in TL after the pandemic and another one that will want to go back to the office):

- the employees that will want to continue working in TL are the ones that are considered to have the best efficiency working in TL and also consider that the TL is affecting the society most of all from an economic and social point of view. In this group, we may consider the employees that are more flexible and adaptable, are working in domains in which many activities can be done in TL, succeed in maintaining a good collaboration and communication with their colleagues. However, they are aware about the economic and social problems that are arising from a TL applied for a long period of time.
- the employees that will not want to continue working in TL are the ones that are considered to have the best efficiency working from the office and also consider that the TL is affecting the society most of all from a psychological and social point of view. In this group, we may consider the employees that are less adaptable, are working in domains in which few activities can be done in TL, and do not succeed in maintaining a good communication with their colleagues. However, they have identified some psychological and social problems that are arising from a TL applied for a long period of time.

**Implications of staying in TL after the pandemic.** The decision of staying in TL after the pandemic is influenced by many factors. From this analysis, the main economic and social factors are work efficiency and satisfaction, the time that is not spent in traffic anymore, the lack of human interaction and the social, economic and psychological aspects that are affecting the society. Applying TL for long term, aspects like these must be taken into consideration: the employer must redesign the ways in which offers support to the employees, how it organize overall the TL and how it takes care of the employees’ mental and physical health; in this regards it is recommended that customized reward packages are created in order to fit to the needs of the employee; the psychological factor that seems to be present and which affects in a negative way the employees—this represents the lack of social communication and also the burnout and high tiredness symptoms. It is possible that elements like these to become mandatory for the employer on the long term for the TL. 

Q4: which are the main profiles for the employees regarding the work efficiency during TL?

The answer for this question in based on the clusters obtained for Q1. In this regard, as answer for Q4, there were identified several profiles of employees and the most representative were analyzed. By analyzing these profiles, it is confirmed that the employees that had the best work efficiency during TL were the ones that were willing to spend more money and to make additional investments and expenses (these aspects are true for 44% from the respondents). On the other hand, the employees for which the work- efficiency was not good at all have encountered problems with the employer, not receiving any help from it during the TL, the specific activities were not suitable for TL, and also they did not make any additional expenses (these aspects are true for 22% from the respondents). 

Therefore, by analyzing these two profiles, we may say that the economic and the financial factors are very important as it is also the involvement of the employer. In many cases, the employer may help the employee to overcome these needs. However, there are also other things that the employer may do, things like: digitalizing the main activities using suitable software, allowing the employee to take the equipment needed at home, offering a bonus to the employee as support, in order to arrange an office in his/her home and also paying licenses for support software, taking more care of the employees’ health. This situation in not very easy for companies because they must make big investment and treat each employee based on his/her needs. On the other hand, a lot of costs were reduced by not having people each day into offices. These economic and financial decisions are hard to take because no company wants to make investments in something that is not useful anymore on the long term.

Q5: which are the main profiles for the employees regarding the work satisfaction during TL?

The answer for this question in based on the clusters obtained for Q2. In this regard, as answers for Q5, there were identified several profiles of employees and the most representative were analyzed. By analyzing these profiles, it is confirmed that the employees that had the best work satisfaction during TL were the ones that have very high work efficiency, consider that the TL had a positive influence over the social communication efficiency and did not need additional hours to complete their activities. The other opposite profile, in which the employees consider having a low satisfaction, consider that the office work is the best way to accomplish their tasks and social communication was affected in a negative way during TL. 

By analyzing these profiles, we may conclude that the work efficiency is strongly related to the work satisfaction and to social communication. In order to improve these aspects, the companies must consider the aspects that are influencing work efficiency (like economic and financial aspects) and to design solutions how to overcome the remote communication issues that some employees encounter. These solutions imply investments and a better understanding of each employee. The communication with the employee must be seen not only in terms of tasks and deadlines, but as a complex and continued support that eliminates the physical distance between the employee & colleagues and the employee & the company overall. In this context, the company must take care of the security aspects, so each employee to be sure that his/her text and video sessions are safe.

Before TL, we may say that all the laws and regulations were, most of them, very clear and the actors involved knew very well what to expect for regarding the management activities in all aspects (economic, social, financial, etc.).

During the TL it was necessary to redesign and to think to the ways in which the activities are done, how the roles and the responsibilities are covered, how the team members interact in order to achieve the greatest work efficiency and work satisfaction as a team, and also as a company. An important aspect in this regard is the support that the company offers to the employees, aspect that influences the others very much. It can be considered the starting point in the relation state-employer-employee. With companies that were forced to change the ways of doing activities and tasks, it can be considered that organizational culture was affected as well. From this point of view, it is hard to say how many things will return to the initial state and how many will remain as they were during TL. 

After the pandemic is gone, it is very likely that the employees working in the public sector to return to office. Regarding the employees that are working in the private sector instead, for them, especially for the ones with jobs suitable for TL, the situation will be different because it is very likely they will continue to want to stay in TL. For them, having a good support from the employer, willing to invest in an office at home, not wanting to spend any more time in traffic and finding it easy to communicate with colleagues are enough arguments for TL. However, for them, some aspects must be clarified, such as: based on the chosen location, the employee must be aware of the accidents that may happen and how the law is covering these accidents and which type of insurance must be used; if the employer is making efforts to cover the expenses of an employee that is in TL, it is very important to know exactly the costs, because in some situations the cost may be higher than the work from office; the employer must think also to the mobility degree of its employees and to the way in which it supervises them, especially when they are located in other countries; the employer must monitor the employee and also it needs to know as much as it can about their activities, but here it is a very thin line between personal data and information, and job data and information—for this reason a mutual relation based on trust and communication must exist between the employer and employee. 

Based on the results obtained, especially the ones from the second experiment, we may conclude that during TL there was a strong relation between work efficiency and work satisfaction. For this reason, the mechanism of effects of TL regarding these two aspects is complex and involves economic and social factors that were analyzed. Let’s consider and start with the aspect that every employer and any employee want to achieve, namely, work efficiency. 

The effects of obtaining a high level of work efficiency are: the employer must develop a very detailed analysis of the activities that are performed, which implies more money to invest (resource allocation—economic factor), then it must identify how much support it can offer to the employees and what kind of support (economic factor), it must identify how it can assure health protection and in the same time how to monitor the employee (economic and social factors); the employee must want to adapt to the new TL conditions (economic factor) and to have good communication with the employer and colleagues (social factor). 

Having all these aspects in mind, we must identify what effects imply a good work satisfaction. These effects are: the companies must try to offer the best work environment for the employees and to offer all the tools that they need (economic factor), to seek good work efficiency from the employees, to have a legal framework very clear and stable, the employees to be informed quickly about the new regulations and laws, to seek what motivates more a certain employee.

Creating a mechanism that accomplishes all the aspects mentioned above is not easy taking into account that: every employee is different and has different needs, how well the employer is able to understand an employee, how quickly the employer offers support in a certain situation, how the new laws and regulations are applied by the employer, and how well the state understands the situations that occurs on the labor market, how well the state can support financially the companies that have difficulties, and so forth. Therefore, there are many aspects that must be covered in order to obtain good work efficiency and a high level of work satisfaction. 

## 7. Conclusions

It is clear that with the onset of the pandemic and TL, many things had to be done differently in a forced manner and time was too short to allow enough time for all actors involved (such as employees, employers and the state) to adapt. This adaptation has come slowly, producing changes in many aspects of the society as a whole.

For this study, a data mining analysis was conducted that comprises five experiments that have revealed interesting data patterns. According to each experiment, the findings are described in a detailed manner in the previous section. The main conclusion for each one is:

- for the first cluster experiment that identifies the relationship between the work efficiency that employees felt that they had during TL and certain social and economic factors—first, the employees that have considered having the best work efficiency, in which case the involvement of all the actors is mandatory at a high level (the employee made investment to transform his/her home, the employer has offered support and the state has created the appropriate legal framework for TL); second, the employees that have considered not having a good work efficiency at all, in which case even they consider that the state has created a good legal framework for TL, they consider also that the employer made no effort to help them and also they were not open to transform their home into an office and to create the right accommodation.
- for the second cluster experiment that identifies the relationship between the work satisfaction degree that employees felt during TL and certain social and economic factors—the employees that have considered having the highest work satisfaction: first, the employees that have considered to have the best work efficiency in TL, in which case they were able to adapt quickly, to understand the situation and to overcome easily the challenges imposed by the TL; second, the employees that have considered having an average work satisfaction and want to return to office as soon as possible, in which case they have considered the office work the best manner to do the job and to accomplish the activities, the TL having a negative effect for the communication between colleagues. What the two groups have in common is that they have considered as the main advantage the time saved from staying in traffic and as main disadvantage the lack of human interaction.
- for the third cluster experiment that identifies the relationship between the impact of teleworking after the pandemic and several factors like works satisfaction and efficiency and some others factors point of view—first, the employees that will want to continue working in TL have considered to have the best efficiency working in TL and also have considered that the TL is affecting the society most of all from an economic and social point of view, the employees from this group are more flexible and adaptable, being able to maintain a good collaboration and communication with their colleagues; second, the employees that will not want to continue working in TL are those that have considered to have the best efficiency working from the office and that the TL is affecting the society most of all from a psychological and social point of view, the employees from this group are less adaptable, are working in domains in which few activities can be done in TL and they are not able to maintain a good communication with their colleagues.
- for the first classification experiment that offers a detailed analysis of the clusters identified based on the work efficiency in the first cluster experiment and highlights the most common profiles of the employees—analyzing the most different two profiles confirms the fact that the employees that had the best work efficiency during TL were the ones that were willing to spend more money and to make additional investments and expenses. The other employees, for which the work-efficiency was not good at all, have encountered problems with the employer, not receiving any help from it during the TL, the specific activities were not suitable for TL, and did not make any additional expenses.
- for the second classification experiment that offers a more detailed analysis of the clusters identified based on the work satisfaction in the second cluster experiment and highlights the most common profiles of the employees—analyzing the different two profiles it is confirmed the fact that the employees that had the best work satisfaction during TL were the ones that have very high work efficiency, TL had a positive influence over the social communication efficiency and did not need any additional hours to complete their activities. The other employees with a low work satisfaction have considered that the office work is the best way to accomplish their tasks and the social communication was affected in a negative way during TL

Personal contribution for this research consists in creating a survey that was sent to employees and based on the answers the data mining analysis was made, the data preprocess, choosing the analysis direction and their suitable attributes, applying the algorithms, obtaining the results and the findings. 

Regarding the impact and significance of TL on the economy and society, the employee must adapt to several aspects such as the way he carries out his activities, the way he presents the results of his activities, the way he collaborates with colleagues and superiors, and also the location. On the other hand, the limited interaction with family members and friends also contributes in a negative way to the mental health of employees. Communication is often a solution of the work problems. Aspects such as the design of an adequate office space inside the house, access to internet services and specific equipment used in TL, the degree of understanding by the family, the number of working hours that often becomes higher than normal working hours, dependence on the silence that characterize the location and the surrounding area, mental and physical health are important.

The employer must also adapt, creating the optimal conditions for the employees. In some domain areas it is harder, in others easier. Here, an important role is given by the ability of companies to understand the employees at least from economic, social, psychological, and emotional point of view. For this, many resources must be allocated, resources like financial resources (bonuses, etc.), material resources (laptops, various devices, etc.) and human resources (specialized people who understand exactly the needs of employees, i.e., psychologists). A big challenge for the employer is to make investments and to know exactly how many resources to allocate considering that in a certain period of time, at least a part of the employees will return to the office. The framework in which the employer can offer support to the employees is largely created by the state. For this reason, the state also plays an important role in influencing the efficiency and satisfaction of the work done by the employee.

After the pandemic, it is difficult to predict whether society’s life will return to what it was before this thing global issue developed. Working from home (or TL) with all the shortcomings and disadvantages it offers and some of which have been mentioned in this analysis, has given employees the opportunity to have very high mobility in terms of work. For this reason, in addition to the fact that they can choose the best location for work, with the most relaxing environment and in which to have maximum efficiency, they can hope for long-distance jobs, which until now they did not even think about.

The most important effects of TL were felt economically and socially. At the level of national and international economy, many fields of activity (such as tourism, services, etc.) have suffered producing effects down to the lowest level of society. Companies had to adapt on the fly and deal with all unforeseen situations in a different way so far. From a social point of view, people felt shortcomings, the human individual being a social being. The lack of communication of many and/or the need to adapt to new ways of socializing made many go out of their comfort zone and learn new things about remote communication.

During the pandemic, even some security health measures were dropped, according to a study made by PricewaterhouseCoopers (PwC) on 300 companies (Georgescu, 2020), as 45% companies will not ask to the employees to return to the offices after the pandemic and 21% are thinking to use a hybrid approach (TL and work office work). Therefore, we can say that during the pandemic, although TL was a forced health measure used to stop spreading the virus, after the pandemic, for some it can become a way of life and for others, who want to return to the office, a viable alternative for the current job.

## 8. Future Development

Data and information collection and the results of the data mining analysis described in this research could be used to identify and to extract knowledge for other several analysis pathways. Having the factors that are already in the dataset, several attributes may be added in order to make the analysis more detailed. For example, more information to be added that describe the TL from the employer’ point of view or from the people that co-habits with the employee. An interesting analysis may consist in finding out if the work efficiency and satisfaction depend also on the people that are in the house while the employee is in TL. Some other algorithms may be used to identify new correlations and patters that exist in the analyzed data.

## Figures and Tables

**Table 1:** Analysis directions.

No.	Analysis	Groups of Used Attributes	Class Attribute	Results of the Analysis and Research Question to Answer
I	Factors that are influencing the work efficiency during TL	A, B, C, D, F, G	E	a. Obtaining a score for the most important social and economic attributes related to the class attribute = > attribute **ranking (Q1)** b. Clustering—grouping the employees based on similarities => **clusters for the employees (Q1)** c. Classification analysis based on cluster assignment => **employees’ profiles related to the group attributes (Q4)**
II	Factors that are influencing the work satisfaction during TL	H, I, E	K	a. Obtaining a score for the most important social and economic attributes related to the class attribute = > **attribute ranking (Q2)** b. Clustering—grouping the employees based on **similarities => clusters for the employees (Q2)** c. Classification analysis based on cluster assignment => **employees’ profiles related to the group attributes (Q5)**
III	Analysis of factors that may change the mentality about TL taking based on the work satisfaction and efficiency during TL	B (activity domain), K, E, J, I (main advantage and main disadvantage)	L	a. Clustering—grouping the employees based on similarities => **clusters for the employees (Q3)**

**Table 2:** Data mining approaches.

No.	Data Mining Approaches	Results of the Analysis and Algorithms Used
I	Clustering (3 experiments)	Obtaining a score for the most important social and economic attributes related to the class attribute => **attribute ranking** (*Gain Ration Attribute Evaluator* and *Ranker Search Method*)
		Clustering—grouping the employees based on similarities => **clusters for the employees** (*Simple K-Means algorithm*)
II	Classification (2 experiments)	Classification analysis based on cluster assignment and a class attribute => **employees’ profiles related to the group attributes** (*PART and J48 algorithms*)

**Table 3:** Cluster analysis of work efficiency.

Attribute	Full Data	Cluster 0	Cluster 1	Cluster 2
	Instances number: 377	165	127	85
Status on labor market (group B)	Employed at a private company	Employed at a private company	Employed at a private company	Employed at a private company
Employed (group B)	Full time	Full time	Full time	Full time
**Efficiency TLW vs. normal activity (group E)**	**Varies based on activity**	**TL best**	**Varies based on activity**	**Normal activity** **is the best**
Employer effort to facilitate TLW (group D)	Yes	Yes	Yes	No
Home accommodation for TLW (group C)	Yes	Yes	Yes	No
Additional investments for TLW (group C)	No	Yes	No	No
Amount of the additional investments for TLW (group C)	No investment	Between 501 and 1000 RON	No investment	No investment
Additional expenses for TLW (group C)	Yes, quite a lot	Yes, quite a lot	Not at all or very few	Not at all or very few
Additional expenses ELECTRICITY (group C)	Yes	Yes	Yes	Yes
Additional expenses TELEPHONY (group C)	No	Yes	No	No
Additional expenses INTERNET (group C)	No	Yes	No	No
Additional expenses WATER and SANITATION (group C)	Yes	Yes	Yes	No
Additional expenses HEATING and AIR CONDITIONING (group C)	Yes	Yes	Yes	No
Estimated expenses generated by TLW (group C)	Under 100 RON	Between 100 and 500 RON	Under 100 RON	Under 100 RON
Information degree about government regulations on TLW (group C)	3	3	4	3
Employer continuously support for TLW (group D)	Sustained	Sustained	Sustained	Not sustained
Regulations impact supporting TLW on your employer (group F)	High impact	High impact	High impact	High impact
TLW appropriate in doing the main activities (group G)	Only part of the main activities	Only part of the main activities	Only part of the main activities	No
Salary level (group B)	Between 2501 and 4000 RON	Between 2501 and 4000 RON	Between 1300 and 2500 RON	Between 2501 and 4000 RON
Last level of studies (group A)	High school	Bachelor	High school	Bachelor
Activity domain (group B)	Economic	Economic	Economic	Services
Company number of employees (group F)	Between 10 and 49	Between 50 and 250	Between 10 and 49	Between 10 and 49
Geographic region (group A)	Bucuresti Ilfov	Bucuresti Ilfov	Sud Muntenia	Bucuresti Ilfov
Age (group A)	Between 40 and 55	Between 26 and 40	Between 18 and 26	Between 40 and 55
Sex (group A)	F	F	F	F

**Table 4:** Cluster analysis of work satisfaction.

Attribute	Full Data	Cluster 0	Cluster 1	Cluster 2
	Instances number: 377	100	149	128
Efficiency TLW vs. normal activity (Group E)	Varies based on activity	TLW has the best work efficiency	Varies based on activity	Normal activity has the best work efficiency
TLW means also additional working hours (Group H)	No	No	Yes	No
TLW positive influence over the communication efficiency (Group I)	No	Yes	No	No
**Satisfaction degree for TLW activity (Group K)**	**Average**	**High**	**Average**	**Average**
Main advantage of TLW (Group I)	Saving time spent in traffic	Saving time spent in traffic	Saving time spent in traffic	Saving time spent in traffic
Disadvantages of TLW (Group I)	Yes	Yes	Yes	Yes
Main disadvantage of TLW (Group I)	Lack of human interaction	Lack of human interaction	Lack of human interaction	Lack of human interaction
Problem of TLW (Group I)	Decreasing the communication level between team members	Decreasing the communication level between team members	Lack of direct contact employees customers	Decreasing the communication level between team members
Income level during the pandemic (Group H)	Remained the same	Increased	Remained the same	Remained the same

**Table 5:** Cluster analysis of impact of teleworking after the pandemic.

Attribute	Full Data	Cluster 0	Cluster 1	Cluster 2
	Instances number: 377	142 (38%)	144 (38%)	91 (24%)
Efficiency TLW vs. normal activity (Group E)	Varies based on activity	TLW efficiency is better	Varies based on activity type	Normal activity efficiency is better
Satisfaction degree for TLW activity (Group K)	Average	Average	Average	Average
Main advantage of TLW (Group I)	Saving time spent in traffic	Saving time spent in traffic	Saving time spent in traffic	Saving time spent in traffic
Main disadvantage of TLW (Group I)	Lack of human interaction	Lack of human interaction	Lack of human interaction	Lack of human interaction
Factors influence POV about TLW 1st place (Group J)	social	economic	social	psychological
Factors influence POV about TLW 2nd place (Group J)	social	social	economic	social
Factors influence POV about TLW 3rd place (Group J)	economic	psychological	psychological	economic
Factors influence POV about TLW 4th place (Group J)	cultural	cultural	cultural	natural
Factors influence POV about TLW 5th place (Group J)	natural	natural	natural	cultural
Activity domain (Group B)	Economic	Other services	Economic	Economic
**TLW after the pandemic** (Group L)	**Yes but only for certain activities**	**Yes full time**	**Yes but only for certain activities**	**No**

**Table 6:** Confusion Matrix.

a	b	c	*<-- Classified as*
150	11	4	*a = cluster0*
11	98	18	*b = cluster1*
9	22	54	*c = cluster2*

**Table 7:** Accuracy of the results.

	TP Rate	FP Rate	Precision	Recall	F-Measure	ROC Area	Class
	0.909	0.094	0.882	0.909	0.896	0.918	cluster0
	0.772	0.132	0.748	0.772	0.76	0.838	cluster1
	0.635	0.075	0.711	0.635	0.671	0.755	cluster2
Weighted Avg.	0.801	0.103	0.798	0.801	0.799	0.855	

**Table 8:** Confusion Matrix.

a	b	c	*<-- Classified as*
83	9	8	*a = cluster0*
7	139	3	*b = cluster1*
4	3	121	*c = cluster2*

**Table 9:** Accuracy of the results.

	TP Rate	FP Rate	Precision	Recall	F-Measure	ROC Area	Class
	0.83	0.04	0.883	0.83	0.856	0.91	cluster0
	0.933	0.053	0.921	0.933	0.927	0.955	cluster1
	0.945	0.044	0.917	0.945	0.931	0.978	cluster2
Weighted Avg.	0.91	0.046	0.909	0.91	0.909	0.951	

## Appendix A

**Table A1:** Attributes groups (TL—Teleworking).

Group ID	Attribute Group	Attributes (Factors)
A	Personal information about the employee (social)	23_A_**Last_level_of_studies** {High school, Bachelor, Master, PhD} 26_A_**Geographic_region** {Bucuresti_Ilfov, Sud_Vest_Oltenia, Nord_Est, Sud_Muntenia, Sud_Est, Centru, Vest, Nord_Vest} 27_A_**Age** {between_18_and_26, between_26_and_40, between_40_and_55, over_55} 28_A_**Sex** {M, F}
B	Information about the employee’s professional activity (social)	01_B_**Status_on_labor_market** {Employed_at_a_public_institution, Employed_at_a_private_company, Entrepreneur, Others} 02_B_**Employed** {Full_time, Part_time, Others} 03_B_**TLW_in_2020** {Yes_Full_time, Yes_Part_time, No} 22_B_**Salary_level** {under_1300_RON, between_1300_and_2500_RON, between_2501_and_4000_RON, between_4001_and_5500_RON, over_5500_RON} 24_B_**Activity_domain** {Agriculture_and_forestry, Constructions, Economic, HR, Industry, Insurance, IT, Others, Services_Defense, Services_Education, Services_Financial_banking, Services_Medical, Services_Others, Services_Public_administration }
C	Home accommodation for teleworking (economic)	06_C_**Home_accomodation_for_TLW** {Yes, No} 07_C_**Additional_investments_for_TLW** {Yes, No} 071_C_**Amount_of_the_additional_investments_for_TLW** {no_investment, no_more_than_500_RON, between_501_and_1000_RON, between_1001_and_2000_RON, over_2000_RON} 08_C_**Additional_expenses_for_TLW** {Yes_quite_a_lot, Not_at_all_or_very_few} 0811_C_**Additional_expenses_ELECTRICITY** {Yes, No} 0812_C_**Additional_expenses_TELEPHONY** {Yes, No} 0813_C_**Additional_expenses_INTERNET** {Yes, No} 0814_C_**Additional_expenses_WATER_and_SANITATION** {Yes, No} 0815_C_**Additional_expenses_HEATING_and_AIR_CONDITIONING** {Yes, No} 082_C_**Estimated_expenses_generated_by_TLW** {under_100_RON, between_100_and_500_RON, between_501_and_1000_RON, over_1000_RON} 15_C_**Information_degree_about_government_regulations_on_TLW** {1,2,3,4,5}
D	Support provided by the employer for teleworking	05_D_**Employer_effort_to_facilitate_TLW** {Yes, No, I_can_not_say} 17_ D_**Employer_continuously_support_for_TLW** {Sustained, Not_sustained, I_can_not_say }
E	Efficiency of teleworking vs. normal activity (economic)	04_E_**Efficiency_TLW_vs_normal_activity** {TLW_best, NormActiv_best, Varies_based_on_activity }
F	Information about the employer/company	18_F_**Regulations_impact_supporting_TLW_on_your_employer** {Very_high_impact, High_impact, Low_impact, Very_low_impact, I_cannot_appreciate} 25_F_**Company_number_of_employees** {under_10, between_10_and_49, between_50_and_250, between_251_and_1000, between_1001_and_5000, over_5000}
G	Employee—telework in relation to basic activities (economic)	21_G_**TLW_appropiate_in_doing_the_main_activities** {All_the_main_activities, Only_part_of_the_main_activities, No}
H	Consequences of teleworking (economic)	09_H_**TLW_means_also_additional_working_hours** {Yes, No} 19_H_**Income_level_during_the_pandemic** {Increased, Decreased, Remained_the_same, Varied}
I	The impact of teleworking (social)	10_I_**TLW_pozitiv_influence_over_the_communication_efficiency** {Yes, No} 12_I_**Main_advantage_of_TLW** { Additional_free_time_obtained, Efficient_management_of_working_hours, Financial_savings, Improving_work_life_balance, Others, Saving_time_spent_in_traffic} 13_I_**Disadvantages_of_TLW** {Yes, No, I_can_not_say} 131_I_**Main_disadvantage_of_TLW** {Limited_access_to_resources, Lack_of_communication_between_employees, Lack_of_concentration, Lack_of_human_interaction, Decreased_productivity, More_allocated_resources, Decreased_salaries} 14_I_**Problem_of_TLW** { Time_delimitation_for_work_and_personal_problems, Limited_access_to_documents, Lack_of_direct_contact_employees_customers, Protection_of_confidential_data, Risk_of_not_advancing_in_the_career, Decreasing_the_communication_level_between_team_members, Others}
J	Factors ranking	161_J_**Factors_influence_POV_about_TLW_1st_place** {cultural, economic, natural, psychological, social} 162_J_**Factors_influence_POV_about_TLW_2nd_place** {cultural, economic, natural, psychological, social} 163_J_**Factors_influence_POV_about_TLW_3rd_place** {cultural, economic, natural, psychological, social} 164_J_**Factors_influence_POV_about_TLW_4th_place** {cultural, economic, natural, psychological, social} 165_J_**Factors_influence_POV_about_TLW_5th_place** {cultural, economic, natural, psychological, social}
K	Satisfaction degree (social)	11_K_**Satisfaction_degree_for_TLW_activity** {Low, Average, High}
L	Impact of teleworking after the pandemic	20_L_**TLW_after_the_pandemic** {Yes_full_time, Yes_only_certain_activities, No}

**Questionnaire**

On the economic and social impact of telework activity in changing the behavior of employees in Romania

1. What is your status on the labor market?

a. Employed in a public institution

b. Employed in a private firm/company

c. Entrepreneur

d. Other status (like liberal professions, etc.)

2. Are you employed:

a. full time

b. part time

c. other

3. In 2020, did you carry out activity in regime of telework?

a. Yes

b. No

c. Partially

4. Do you consider that you performed the telework activity with the same efficiency as at the office?

a. work efficiency is the best during TLW

b. work efficiency varies based on the activity type

c. work efficiency is the best during normal activity

5. Do you consider that your employer has made a sustained effort to facilitate your telework activity?

a. Yes

b. No

c. I don’t know/I don’t pronounce

6. Do you think that you have found at home an environment conducive to the performance of work tasks?

a. Yes

b. No

7. Did you have to make additional investments to perform the tasks and service activities for telework?

a. Yes

b. No.

7.1 Amount of the additional investments for TLW (500 RON are approx.. 100 Euro)

a. no investment

b. no more than 500 RON

c. between 501 and 1000 RON

d. between 1001 and 2000 RON

e. over 2000 RON

8. Do you incur additional expenses in the current performance of tasks and service activities in telework?

a. Yes, quite a lot

b. No at all or very few

9. For you, as an employee, did telework also require overtime?

a. Yes

b. No

10. Do you consider that in the long run telework will positively influence the efficiency of communication between employer and employees?

a. Yes

b. No.

11. What is the degree of satisfaction you feel in terms of your work in telework?

a. Low

b. Medium

c. High

12. In the situation where you are employed and work in telework, what advantage do you consider to be the most significant? (only one answer shall be chosen)

a. saving time spent in traffic;

b. financial savings achieved;

c. improving the work-life balance;

d. additional free time obtained;

e. efficient management of working hours.

f. other—field with text entered by the respondent

13. Have you identified disadvantages of TLW?

a. yes

b. no

c. I cannot say

13. 1 Which are the main disadvantages of the TLW that you have identified?

a. limited access to resources

b. lack of communication between employees

c. lack of concentration

d. lack of human interaction

e. decreased productivity

f. more allocated resources

g. decreased salaries

14. In the situation where you are employed and work in telework, what problem you encountered do you consider to be the most important? (only one answer shall be chosen)

a. Time delimitation for work and personal problems

b. Limited access to documents

c. lack of direct contact employees—customers

d. protection of confidential data

e. risk of not advancing in the career

f. decreasing the communication level between team members

g. others

15. Mark on a scale of 1 to 5 the extent to which you are informed about government regulations on telework: 1—I am not informed at all, 5—I am very informed

1 2 3 4 5

16. Do the factors considered determine the change of mentalities regarding telework? (First, most important)

a. Social;

b. Economic;

c. Cultural;

d. Psychological;

e. Natural (environmental).

17. Is the telework activity supported by your employer? (one answer)

a. Yes, it is supported

b. No, it is not supported

c. I don’t know/I can’t pronounce

18. How do you assess, on the whole, the impact of work in telework regulations for your employer? (one answer)

a. very high impact

b. high impact

c. low impact

d. very low impact

e. I cannot appreciate

19. During your work in telework, your income level:

a. increased

b. decreased

c. remained the same

d. varied

20. After the pandemic, would you like to continue working in telework?

a. yes, integrally

b. yes, but only certain tasks or activities

c. no

21. Do you consider that telework is appropriate in your field of activity?

a. for all the main activities

b. only for part of the main activities

c. no

21 bis. If there are issues that were not covered in the previous questions and that you would like to report, please present them here:

Information about the respondent

22. What is your salary level (net salary in RON)?

a. under 1300

b. 1.300–2.500

c. 2.501–4.000

c. 4.001–5.500

d. over 5.500

23. What is the last level of studies completed?

a. High school

b. Bachelor

c. Master studies

d. PhD studies

24. What is the field in which you work/carry out activity?

a. Agriculture, hunting, forestry

b. Constructions

c. Economic

d. Human Resources

e. Industry

f. Insurance

g. IT

h. Defense service

i. Education service

j. Financial and banking services

k. Medical service

l. Public administration service

m. Other services

n. Other

25. What is the number of employees in your company?

a. less than 10

b. 10–49

c. 50–250

d. 251–1000

e. 1001–5000

f. over 5000

26. Region of development you come from:

a. Bucharest—Ilfov;

b. South-West Oltenia;

c. North-East

d. South—Muntenia;

e. South-East;

f. Centre

g. West;

h. North-West;

27. What age group do you belong to?

a. 18–25 years

b. 26–40 years

c. 41–55 years

d. over 55 years

28. What is your gender?

a. Male;

b. Female.

**1. What is your status on the labor market?**	**Count**
a. Employed in a public institution	119
b. Employed in a private firm/company	228
c. Entrepreneur	6
d. Other status (like liberal professions, etc.)	24
	**377**

**2. Are you employed:**	**Count**
a. full time	284
b. part time	86
c. other	7
	**377**

**4. Do you consider that you performed the telework activity with the same efficiency as at the office?**	**Count**
a. work efficiency is the best during TLW	105
b. work efficiency varies based on the activity type	154
c. work efficiency is the best during normal activity	118
	**377**

**5. Do you consider that your employer has made a sustained effort to facilitate your telework activity?**	**Count**
a. Yes	193
b. No	104
c. I cannot say	80
	**377**
**22. What is your salary level (net salary in RON)?**	**Count**
a. under 1300	29
b. 1.300–2.500	106
c. 2.501–4.000	137
c. 4.001–5.500	57
d. over 5.500	48
	**377**
**23. What is the last level of studies completed?**	**Count**
a. High school	141
b. Bachelor	139
c. Master studies	93
d. PhD studies	4
	**377**
**24. What is the field in which you work/carry out activity?**	**Count**
a. Agriculture, hunting, forestry	18
b. Constructions	14
c. Economic	91
d. Human Resources	7
e. Industry	27
f. Insurance	18
g. IT	36
h. Defense service	3
i. Education service	29
j. Financial and banking services	35
k. Medical service	4
l. Public administration service	4
m. Other services	75
n. Other	16
	**377**
**25. What is the number of employees in your company?**	**Count**
a. less than 10	52
b. 10–49	109
c. 50–250	106
d. 251–1000	61
e. 1001–5000	30
f. over 5000	19
	**377**
**26. Region of development you come from:**	**Count**
a. Bucharest—Ilfov	171
b. South-West Oltenia	15
c. North-East	26
d. South—Muntenia	101
e. South-East	47
f. Centre	14
g. West	2
h. North-West	1
	**377**
**27. What age group do you belong to?**	**Count**
a. 18–25 years	125
b. 26–40 years	114
c. 41–55 years	128
d. over 55 years	10
	**377**
**28. What is your gender?**	**Count**
a. Male	126
b. Female	251
	**377**
